# Transcriptome analysis of lateral buds from *Phyllostachys edulis* rhizome during germination and early shoot stages

**DOI:** 10.1186/s12870-020-02439-8

**Published:** 2020-05-24

**Authors:** Yuting Shou, Yihua Zhu, Yulong Ding

**Affiliations:** 1 Co-Innovation Center for Sustainable Forestry in Southern China, Nanjing Forestry University, Nanjing, 210037 Jiangsu China; 2 Bamboo Research Institute, Nanjing ForestryUniversity, Nanjing, 210037 Jiangsu China

**Keywords:** *Phyllostachys edulies*, Rhizome lateral bud, RNA-Seq, Alternative splicing, Phytohormone, GA, DELLA protein

## Abstract

**Background:**

The vegetative growth is an important stage for plants when they conduct photosynthesis, accumulate and collect all resources needed and prepare for reproduction stage. Bamboo is one of the fastest growing plant species. The rapid growth of *Phyllostachys edulis* results from the expansion of intercalary meristem at the basal part of nodes, which are differentiated from the apical meristem of rhizome lateral buds. However, little is known about the major signaling pathways and players involved during this rapid development stage of bamboo. To study this question, we adopted the high-throughput sequencing technology and compared the transcriptomes of Moso bamboo rhizome buds in germination stage and late development stage.

**Results:**

We found that the development of Moso bamboo rhizome lateral buds was coordinated by multiple pathways, including meristem development, sugar metabolism and phytohormone signaling. Phytohormones have fundamental impacts on the plant development. We found the evidence of several major hormones participating in the development of Moso bamboo rhizome lateral bud. Furthermore, we showed direct evidence that Gibberellic Acids (GA) signaling participated in the Moso bamboo stem elongation.

**Conclusion:**

Significant changes occur in various signaling pathways during the development of rhizome lateral buds. It is crucial to understand how these changes are translated to *Phyllostachys edulis* fast growth. These results expand our knowledge on the Moso bamboo internodes fast growth and provide research basis for further study.

## Background

Plant vegetative growth is a complicate developmental process which is a combined result of the plants genetic program and various environmental factors [1]. A systematic understanding the mechanism of plant growth offers researchers and farmers the key to increasing crop yields and coping with hazardous agricultural situations. The Moso Bamboo (*Phyllostachys edulis*) is an excellent model to study plant growth [2]. The moso bamboo has great nutritious and economic value, but it is particularly of interest to researchers due to the ability to grow rapidly [2].

Decades of studies have been focused on the general mechanisms of bamboo fast growth [3–7], observing sequential elongation of internodes from the bottom of the culm to the top. The fast growth of Moso bamboo results from the expansion of meristem, which differentiates in the rhizome lateral buds [8, 9]. The appearance of rhizome lateral bud increases the survival rate by providing extra advantages for obtaining nutrients and light in the extremes environment [10]. Thus, bamboo rhizome lateral bud meristem plays a significant role in bamboo morphogenesis.

The development of the rhizome lateral bud into the upright stem relies on the environment, the parent shoot, and the rhizome apex [11]. Environmental factors such as nitrogen content and water condition in soil regulate the growth of lateral buds into shoots [12–15]. The effects of carbohydrate metabolism on rhizome lateral bud shooting are the response to light exposure [16]. By using rice cross-species microarray hybridization, several genes were found to be closely related to the development of *Phyllostachys praecox* rhizome lateral bud, including hormone signaling factor HB1, and CLV1, a signaling peptide in meristem development [17]. Multiple transcriptional factors have been implied to play roles during rhizome bud development [17–20]. Nevertheless, the regulation network of rhizome lateral bud development still remains unknown.

Plant hormones are major regulators of plant growth and development, and are extensively studies in model organisms, such as *Arabiodopsis* and *Oryza*. Various hormones (auxin, Brassinolides and Gibberellic Acids) signaling pathways genes were regulated in different trends or stages in grass rhizome later bud development [21]. In bamboo, the hormone-mediated signaling pathway was one of the major signal transduction regulation in fast-growing culms [22]. Effects of various phytohormones on the development of Moso bamboo were recently reported on transcriptome levels [23–25].

Despite the importance of understanding the molecular mechanism of the rhizome lateral bud development, most previous studies only focused on the up-grounded shoot. In bamboo morphogenesis, the long-term asexual reproduction of rhizome bud controls the rise and fall of the bamboo forest [8]. Moreover, as the initial development stage in the whole bamboo lifecycle, the rhizome lateral bud development progress has lots of unknown details.

Bioinformatics has been a useful and prevalent tool for non-classic model plant researches. However, researches on *Phyllostachys edulis* were hinged and lagged behind due to the lack of technical support in extracting DNA, RNA or proteins from bamboo. It was only until recently that Moso bamboo genome draft was sequenced, bringing opportunity to study the molecular regulation of functional genes in Moso bamboo in a more convenient and meticulous way without reads assembling [22]. Peng et al. used the Illumina sequencing platform to sequence the Moso bamboo shoot and culm after leaf expansion and looked for key regulating factors which control the bamboo fast growing characteristics [22]. He et al. implemented RNA-seq with microscopy to analyze the mRNA and microRNA expressions in the rapid growth of developing culms in Moso bamboo [26].

Alternative Splicing (AS) events lead to the diversification of protein structures and creation of novel functions to benefit the organism or can be associated with genetic diseases [27–29]. In plants, AS has significant influence in plant growth, development and defense, by changing domain architectures of some important proteins. Loss of domains by alternative splicing promoted functional shifts of some auxin response factors [30]. A splicing variant of JASMONATE ZIM-domain protein (JAZ10.4), which lacked Jas domain, and could attenuate signal output in the presence of JA [31]. As an organism with vast intron-containing genes, *Phyllostachys edulis* is no exception to AS events. Almost half of the annotated genes in the recently published reference genome contain AS variants [32]. Those AS events also varies in different tissues [33], growth stages [34] and respond to changes in hormones and environment [34, 35]. Single nucleotide polymorphisms (SNPs) and nucleotide insertion and deletions (Indels) are natural occurring genetic variations that associated with disease, genetic traits and gene evolution. SNPs were used as markers to study the relationship between temperate bamboo species [36]. A systematic review of SNPs and Indels in bamboo related transcriptome research is of dire need.

In this study, we sequenced the transcriptomes of underground samples of rhizome lateral buds in germination stage and early shoot stage. We reported the discovery of novel genes, AS events, SNPs and Indels, which complemented the current annotations in bamboo genome. We compared the transcriptomes of the two stages and investigated how the dynamics of transcription factors, meristem development, carbohydrate metabolism and hormone signaling change. We further verified our transcriptome analysis results by investigating the role of GA in bamboo fast growth. Together, our study could shed new light on the regulation mechanism of Moso bamboo rhizome lateral bud development.

## Results

### Reads mapping and analysis

**Fig. 1:**
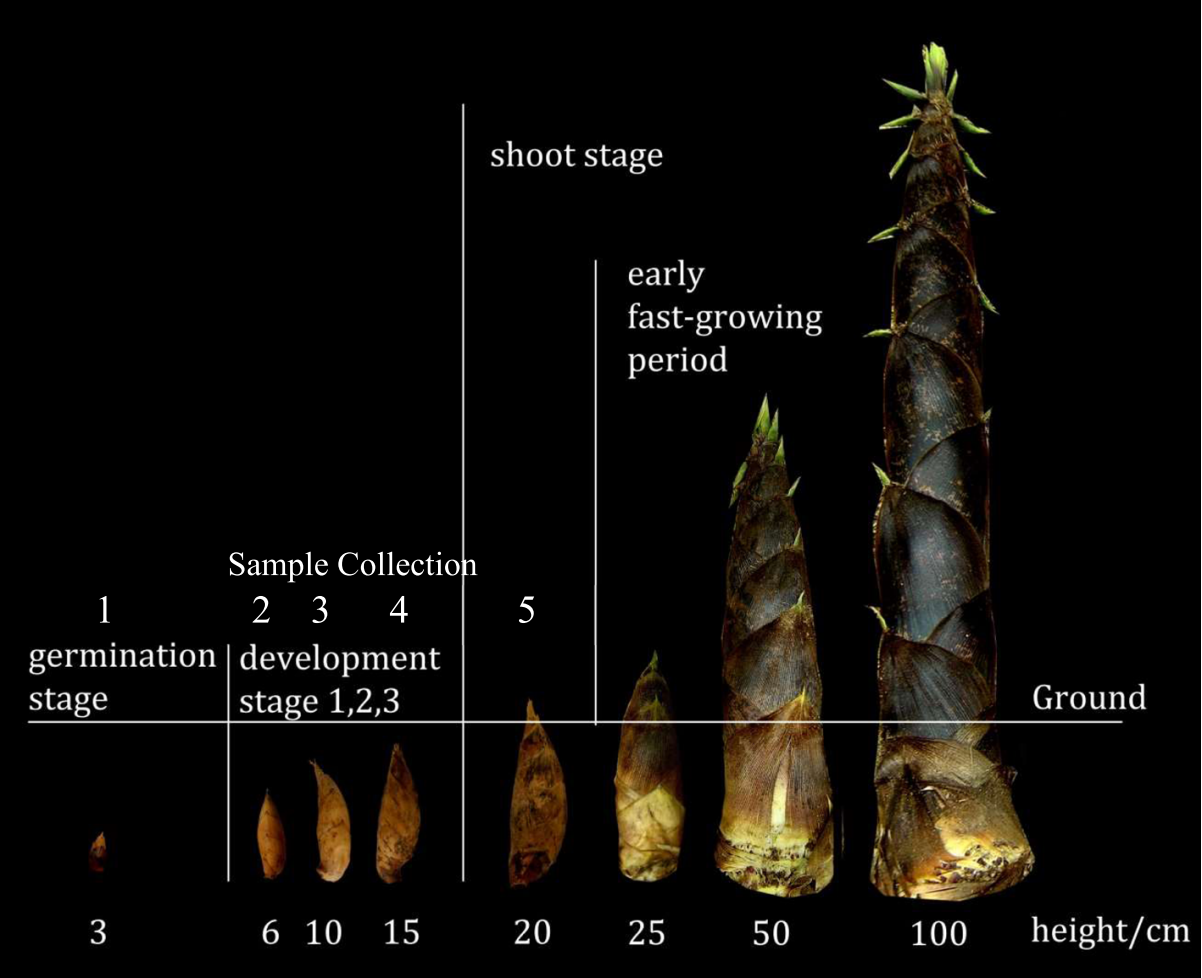
*Phyllostachys edulis* rhizome bud and shoot in different development stages. Stages of *Phyllostachys edulis* rhizome bud were characterized by the bud or shoot length. The samples collected for this study (MSAJ-01 ~ MSAJ-05) are germination stage (MSAJ-01), development stage 1 (MSAJ-02), development stage 2 (MSAJ-03), development stage 3 (MSAJ-04) and shoot stage (MSAJ-05)

The bamboo shoot development can be divided into six stages: dormancy, germination, development stage I, II and III, and shoot stage [37, 38] (Fig. 1). To characterize and generate the expression profiles for rhizome bud development, cDNA samples from rhizome lateral bud of Moso Bamboo in germination stage or early shoot stage were prepared and sequenced.

High throughout RNA-seq reads were mapped onto the published Moso bamboo draft genomic sequences [22] (Table 1). With a pre-treatment to the reads generated, there were 11,863,411 merged single reads and 3,885,776 paired-end reads in germination stage sample, and 20,854,206 merged single reads and 6,910,356 paired-end reads in shoot stage sample. Among them, 14,769,297 (94.9%) reads in germination stage sample and 25,909,031 (93.3%) reads in shoot stage sample can be mapped onto the genome sequences. Only 32.6% of mapped reads in germination stage sample and 31.9% of mapped reads in shoot stage sample were mapped onto the previously annotated genome regions.

**Table 1:** Number of mapped genes from the two rhizome bud development stages

Genome mapping	Germination sample	Shoot sample
Total mapped reads	14769297	25909031
Perfect match	10102246	17621789
Unique match	12150461	21133012
Multi-position match	2618836	4776019
Total unmapped reads	979890	1855531

**Fig. 2:**
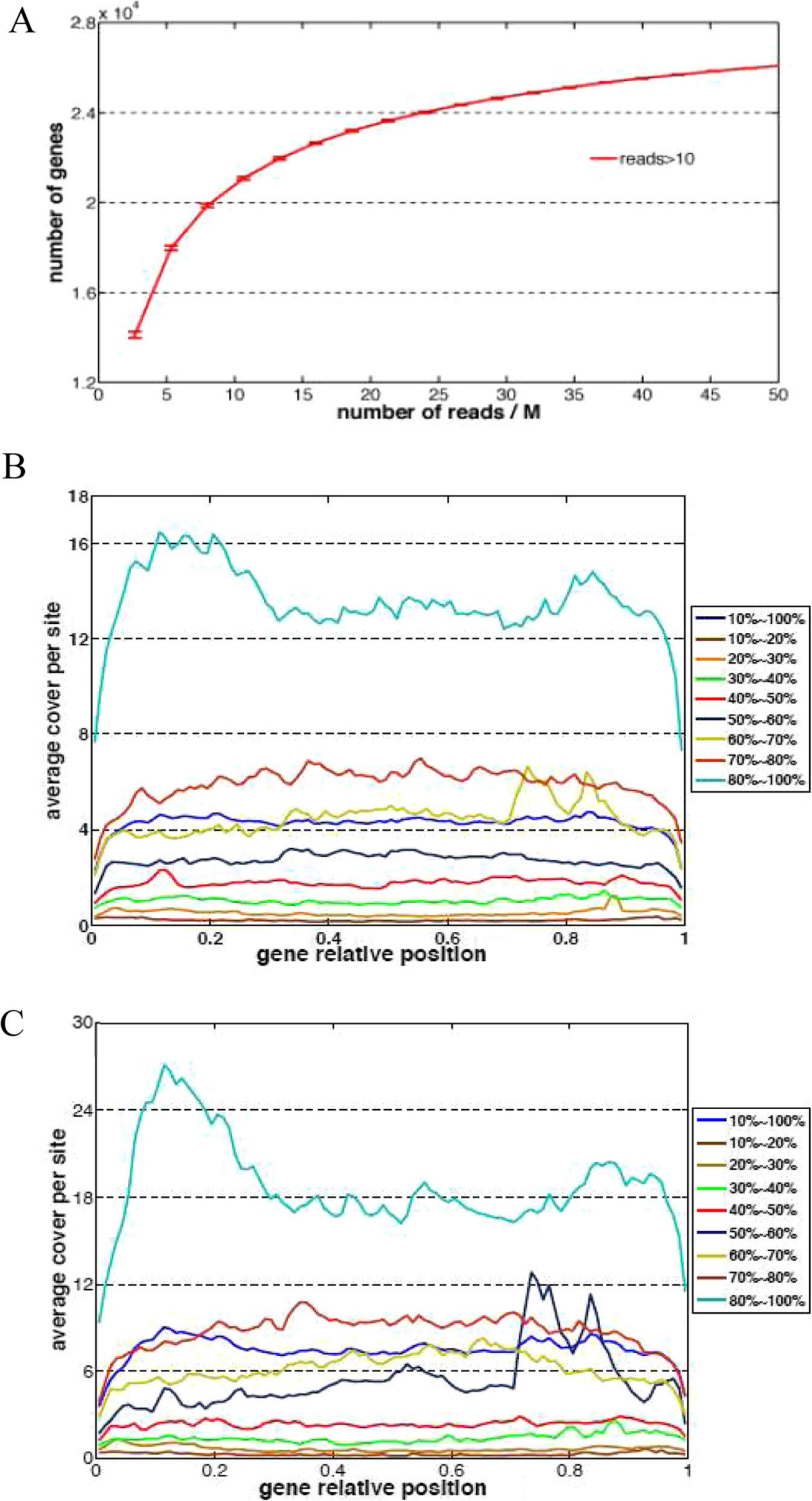
Transcriptome sequence reads analysis. **a** Sequencing Saturation Analysis. As more reads (x axis) were sequenced, the discovery of genes with more than 10 reads reached plateau. **b** and **c** Average reads coverage in gene relative position in germination stage (**b**) and in early shoot stage (**c**). The x axis represented relative position in gene sequence from start ‘0’ to end ‘1’. The y axis showed the average read coverage for each gene position. The genes are grouped according to the total reads coverage from 10 to 100%, as is shown on the right

We thus used Cufflinks to reannotate novel genes by integrating information on reads mapping patterns and previous annotation and obtained a total list of 55,869 genes [39]. Accordingly, 13,939,159 reads (94.4%) in germination stage sample and 24,674,009 reads (95.2%) in shoot stage sample were mapped to these genetic regions, indicating that the reannotation covered the vast majority of reads. In gene saturation line (Fig. 2a), the increase of newly covered genes reached plateau as more reads were sequenced, which indicated that our sequencing depth was high enough to cover most genes expressing in our samples. The average reads covered per site of gene relative positions showed that although a part of sequences located to both terminates of genes were lost, our data had no obvious 5 prime or 3 prime bias (Fig. 2b and c).

### Identification of novel genes in RNA-seq

**Fig. 3:**
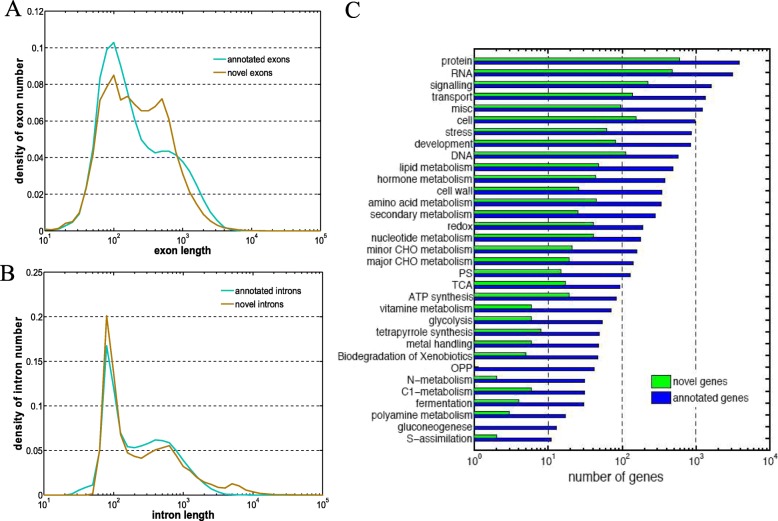
Summary of the features of novel genes. **a** and **b** Distribution of exon lengths (**a**) and intron lengths (**b**) for novel genes (brown) and previously annotated genes (green). **c** Distributions of genes in each functional category using Mapman. novel genes (green) and annotated genes (blue)

We identified 22,107 novel genes (25,808 transcripts or 60,454 exons) within inter-genetic regions with an RPKM cutoff of 1.0 and compared the distributions of exon and intron length between the novel genes and previously annotated genes from published bamboo genome (Fig. 3). Both novel and annotated genes shared a similar distribution of exon length with two peaks at about 200 bp and 700 bp, consistent with recent data. However, there were larger gene density at 700 bp in our newly identified genes due to a proportion of single long exon genes (Fig. 3a). The distribution of intron length in novel genes and annotated genes were also similar, with a high sharp peak at about 100 bp and a low peak at about 500 bp (Fig. 3b). Such similarity in both exon and intron length between novel genes and old genes confirmed the validity of our newly identified genes.

Novel genes were grouped according to the number of exons, with 5770 multiple exon genes (MEG) and 16,337 single exon genes. In MEG set, Trinity predicted 5986 non-redundant coding sequences [40], 5149 of which can be aligned to and annotated by *Oryza sativa japonica* genomic data with an e-value less than 1e-5. We clustered 3153 newly annotated MEG and 23,213 previously annotated genes in Mapman. In each category, the number of newly annotated MEG were proportional to the number of previously annotated genes (~ 10%, Fig. 3c). The majority of newly annotated MEG were involved in basal metabolism, protein and RNA synthesis and cell signaling. In addition, novel genes were identified in categories that were closely related to meristem development such as cell wall (26 genes), cell division and organization (77 genes) and hormone metabolism [41].

In the single exon genes set, 7962 protein sequences were predicted by Trinity [40] and 1737 non-coding RNA sequences were found by blast against Rfam [42], including 1055 rRNA, 58 tRNA, 12 micro-RNA and 37 snoRNA (Table 2). The results above indicated that the sequences identified in this study captured the abundance of novel genes (both functional proteins and non-coding RNA) expressed in Moso bamboo rhizome lateral buds and early shoots. For most plants, only less than ~ 37% of the genes are intronless [43, 44]. The MEG set was considered more reliable. Thus, we continued with MEG set for further functional analysis.

**Table 2:** The number of noncoding RNA

Plant_SRP	rRNA	RNase_MRP	miRNA	tRNA	snRNA	other
7	1055	2	12	58	37	566

### Identification of alternative splicing (AS) events

We also used our data to identify AS events in bamboo. A total of 28,217 AS events were identified by Astalavista-3.2 in the two samples [45] (Table 3), a number similar to previous study on AS events in rhizome bud tissue using PacBio technology [46]. The intron retention events (IR), alternative 3′ splice sites events (Alt 3′ ss), exon skipping events (ES) and alternative 5′ splice sites events (Alt 5′ ss) constituted the most frequent AS events types, which also consistent with previous findings [32, 33]. About 37.2.% and 32.2 of the AS junctions were previously annotated splicing sites in germination stage sample and shoot stage sample, respectively, while 38.0 and 40.6% were assigned into unknown splicing sites in annotated genes. The rest 24.9 and 27.2% junctions were located in novel genes. Together, we identified a total of 10,588 AS related splicing variants. Nine thousand two hundred sixty-three variants originated from ~ 40% of the annotated genes from previously published bamboo genome [22], whereas the rest 1325 were from the novel genes.

**Table 3:** AS events in Moso bamboo underground development stage

Annotation	NO. of Events	AS-code by Astalavista
Intron retention	6677	0, 1^2–
Alternative 3' splicing site	5432	1–, 2–
Exon skipping	4771	0, 1–2^
Alternative 5' splicing site	3934	1^, 2^
Exon skipping1 + exon skipping2	813	0, 1–2^3–4^
Intron retention1 + intron retention2	626	0, 1^2–3^4–
Exon skipping + alternative 3' splicing site	484	1–2^4–, 3–
Alternative 3' splicing site + alternative 5' splicing site	465	1^3–, 2^4–
Mutually exclusive exon	441	1–2^, 3–4^
Exon skipping + alternative 3' splicing site	406	1–2^3–, 4–
Exon skipping + alternative 5' splicing site	395	1^, 2^3–4^
Alternative 5' splicing site + alternative 3' splicing site	391	1^4–, 2^3–
Exon skipping + alternative 5' splicing site	327	1^3–4^, 2^
Alternative 5' splicing site + alternative 3' splicing site	258	1^2–, 3^4–

**Fig. 4:**
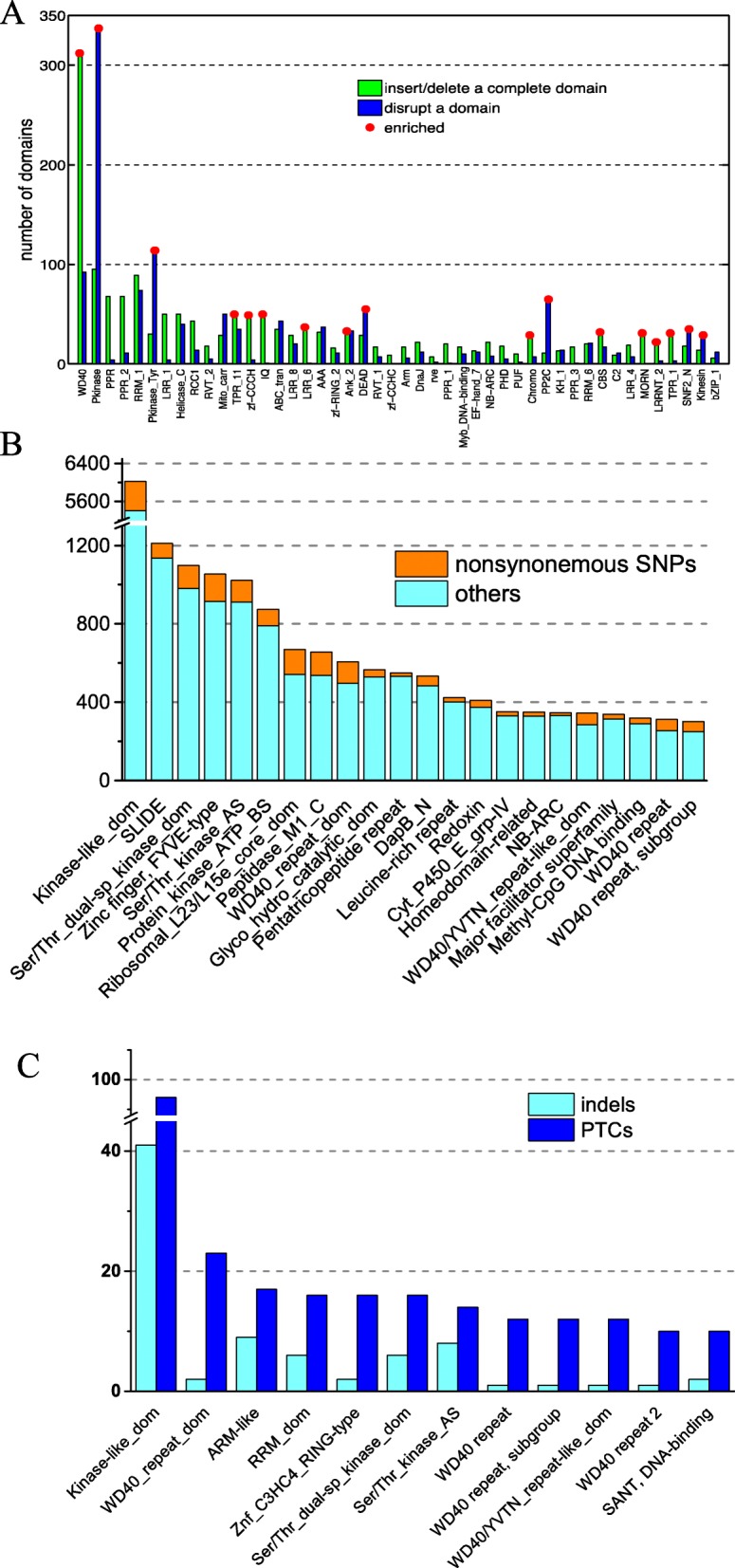
Functional enrichment analysis of AS events and genetic variations discovered during transcriptome analysis. **a** Distrbution of Class I and Class II AS event in different domain families. Class I, blue; Class II, green. Red dot, enriched **b** and **c** Distribution of nonsynonymous mutation (**b**) and coding frameshift indels and premature termination codons (PTCs) (**c**) in protein domain family

AS creates protein isoforms and leads to increased protein complexity and functional diversity. A large portion of AS events in winter bamboo shoot development lead to domain lost in hormone associated genes, potentially regulating hormone signaling [34]. Wang et al. divided alternative splicing events into two classes according to their impacts on domain architectures [47], altering the length of domain (Class I) and retaining/deleting the domain (Class II). To investigate if any protein families ‘prefer’ AS during RNA transcription in Moso Bamboo, we performed clustering analysis to our data. Proteins in PKinase and PKinase_Tyr, DEAD and PP2C families had enriched Class I AS, whereas WD40, TPR_11, IQ and zf-CCCH domain families were prone to Class II AS (Fig. 4a).

### Identification of single nucleotide polymorphisms (SNPs) and base insertions and deletions (Indels)

We next examined single-nucleotide polymorphisms (SNPs), and insertions and deletions (indels) presented in our data. There were 18,602 homozygous SNPs and 25,979 heterozygous SNPs compared to the draft genome. 44.2% homozygous SNPs and 67.8% heterozygous SNPs located at the coding regions of previously annotated genes. We observed more nonsynonymous SNPs (5019) than synonymous SNPs (3208) in homozygous SNPs, while the opposite was seen in heterozygous SNPs (10415 synonymous SNPs and 7206 nonsynonymous SNPs). The nonsynonymous SNPs were more frequently seen in proteins with PKinase domains, WD40 domains, nucleic acid-binding domains and Zinc finger FYVE domains (Fig. 4b).

We have also identified 6544 homozygous indels and 15347 heterozygous indels. Contrary to SNPs, only 5.34% (350) homozygous indels and 11.8% (1811) heterozygous indels located at annotated coding regions. It was also of notice that most of these indels (268 homozygous and 1430 heterozygous) did not cause frame shifts. After function clustering, we found that indels were enriched in protein kinases with ARM-like or RRM-dom domains (Fig. 4c). Premature termination codons (PTCs) often originate from nonsynonymous SNPs or frameshift. It is thus not surprising that protein families enriched in nonsynonymous SNPs and indels also possess PTC more frequently (Fig. 4c).

Genes with two or more nonsynonymous alleles are highly variant genes. Only less than 10% (1518) genes in our RNA-seq data were highly variant, suggesting a close relationship between the sequenced transcriptome and draft genome. These genes are enriched in processes of transportation, hormone metabolism, nucleotide metabolism, minor carbohydrate metabolism and photosynthesis, especially in ABC transport and multidrug resistance system and Jasmonate signaling pathway, indicating a faster gene evolution in these categories.

### Functional analysis on differentially expressed genes

We next investigated how gene expression was differentiated between germination stage and shoot stage. With a RPKM cutoff of 1.0 (both RPKM values of genes in the two samples should be larger than the cutoff), we looked at how major pathways in plant growth change between the two stages (Fig. S1).

#### Transcription factors

**Fig. 5:**
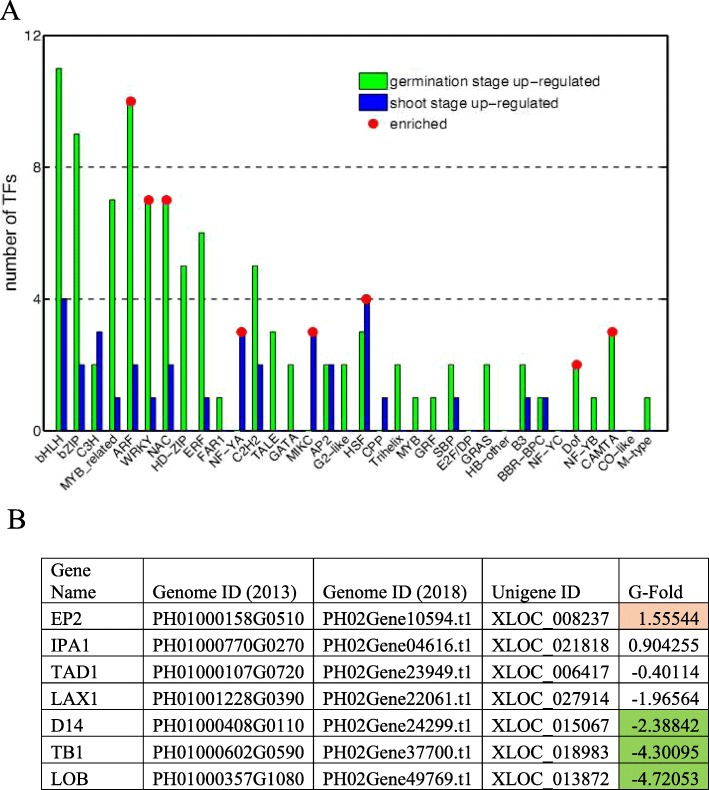
Activities of transcription factor families and axillary meristem development at germination and early shoot stage. **a** Distribution of putative transcription factors upregulated in germination (green) and early shoot (blue) stages. Red dot, the family is enriched in respective stage. **b** Differential expression of putative regulators in axillary meristem development. Green, significantly increased expression at germination stage; brown, significantly increased expression at early shoot stage

Transcription factors regulate gene expression through binding to cis elements in response to environmental and developmental changes. It was estimated by Plant Transcription Factor Database that Moso Bamboo has about 1768 transcription factors from 54 families. We found 138 transcription factors in 35 families displaying expression level changes between germination stage and early shoot stage. As high as three quarters of the transcription factors were highly expressed in germination stages including ARF, WRKY, NAC and DOF families, and the rest quarter transcription factors were highly expressed in early shoot stage, including NF-YA, HSF and MIKC type MADS families (Fig. 5a).

#### Rhizome meristem development

The underground development of the rhizome buds determines the size and yield of mature bamboo. The apical meristem maintains cell proliferation and differentiation for stem elongation and the auxiliary meristem differentiates and develops to branches, leaves and sheaths. We found several negative regulators of auxiliary meristem development LAX1, D14 and LOB were highly expressed during germination stage whereas genes promoting auxiliary meristem development (IPA1 and EP2) were upregulated during shoot stages (Fig. 5b). Our data suggested that auxiliary meristem development was inhibited during germination and promoted during shooting stage.

#### Carbohydrate metabolism

**Fig. 6:**
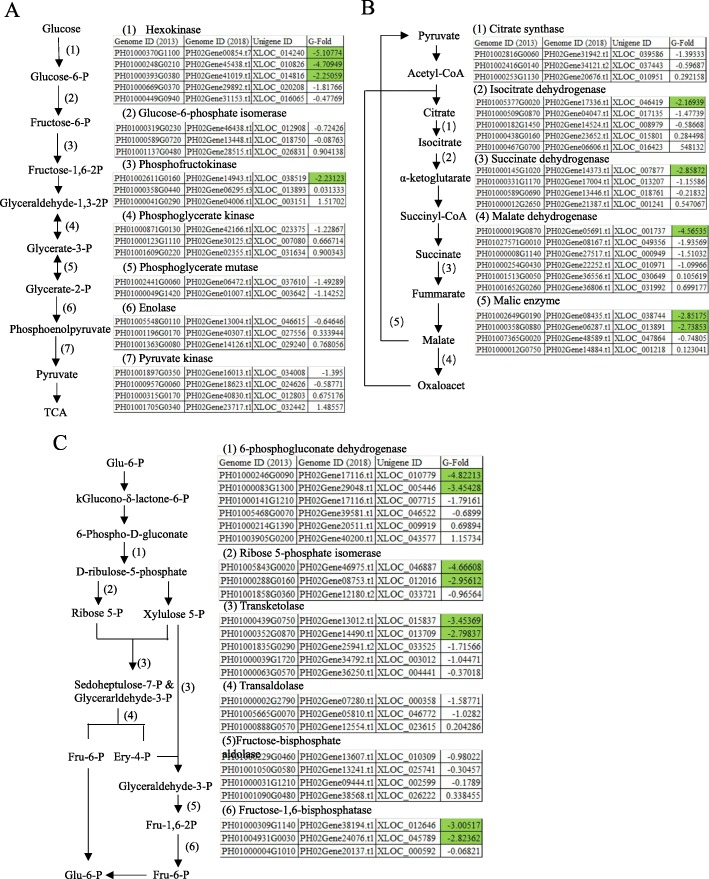
Changes in putative carbohydrate metabolism pathway gene expressions in two development stage. Key players in EMP (**a**), TCA (**b**) and PPP (**c**) pathways were investigated for their expression in germination and early shoot stage. The G-fold number presented how the expression levels in germination compared to early shoot. Green, G-fold < − 2

The rhizome buds are heterotrophs during the germination and early shoot stages, consuming the energy from metabolizing carbohydrates synthesized in above-ground tissues. We compared the gene expressions of the three main carbohydrate metabolism pathways in the two rhizome-bud developmental stages, glycolysis, tricarboxylic acid (TCA) cycle and pentose phosphate pathway (PPP) (Fig. 6).

Glycolysis is the major pathway for glucose metabolism in rhizome bud cells. The phosphorylation of glucose by hexokinase is the first rate-limiting and irreversible step, which initiates glycolysis. The resulting glucose-6-phosphate (G6P) is transformed to fructose-6-phosphate before going through another phosphorylation by ATP-dependent phosphofructkinase (PFK). Finally, a third rate-limiting kinase, pyruvate kinase, transfers the phosphate group from phosphoenolpyruvate to ADP and produces ATP and pyruvate, a TCA cycle precursor. We observed all six putative hexokinase related sequences were greatly expressed at germination stage and three of them were more than 2 G-fold over early shoot stage (Fig. 6a). Other two rate-limiting kinases also saw strongly (PFK, XLOC_038519, − 2.23123) and moderately (pyruvate kinase, XLOC_034008, − 1.395) increased activity during germination stage.

The pyruvate produced from glycolysis can form acetyl-CoA and go through a series of oxidization in TCA cycle, the most effective energy-producing pathway. We noted that a majority of putative genes in all steps of TCA cycle were upregulated during germination stage (Fig. 6b). A similar trend was also observed in PPP pathways, as the gene expression were elevated during germination stage, including the rate-limiting 6-phosphogluconate hydrogenase (Fig. 6c). Thus, the carbohydrate metabolism appeared more active during bud germination compared to early shoot development.

#### Hormone

Plant hormones play vital roles in the growth of bamboo shoot. We found putative genes annotated to indole-3-acettic acid (IAA), Brassinosteroid (BR) and gibberellin acid (GA) synthesis and signal transduction pathway displayed significant expression level changes during Moso Bamboo rhizome bud underground development.

**Fig. 7:**
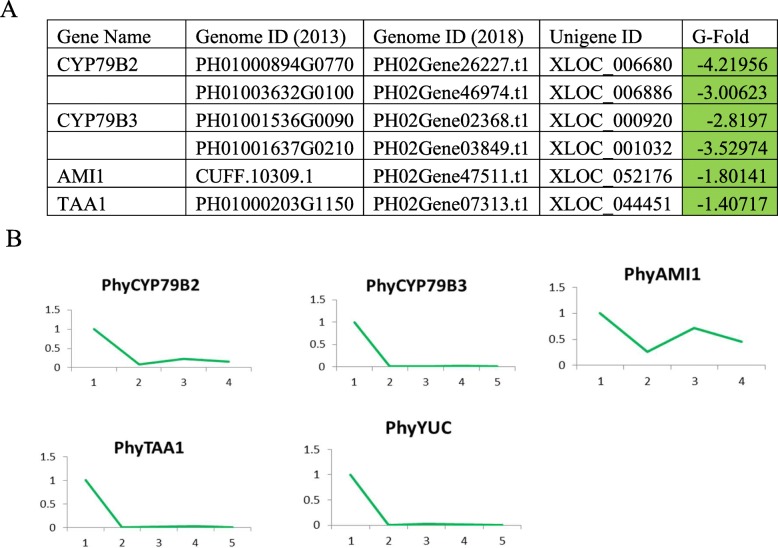
Changes in expression of putative IAA synthesis pathways genes. **a** Transcriptome analysis of putative genes related to auxin synthesis. Green, significantly increased expression at germination stage. **b** Relative expression of putative genes in (**a**) at various underground stages of rhizome bud development. Stage 1 ~ 5 are germination, development stage 1,2 and 3 and early shoot stage, respectively. For CYP79B2 and AMI1, only the first four stages were assayed due to sample availability. The expressions were measured by qPCR and results were normalized based on the germination stage (stage 1) data

IAA, the main auxin produced in plants, participates in cell proliferation, cell cycle regulation, cell elongation and cell wall development of the apical meristem. Using Trptophan as a precursor, IAA can be synthesized via multiple routes (Fig. 7). We found decreased expression level in putative gene annotated to major IAA synthesis pathways (CYP79B2 and CYP79B3 in IAOx route, IAM1 in IAM route and TAA1 in IPA route). The decline was further confirmed using reverse transcription qPCR to measure the expression of those putative genes throughout the five underground development stages of Moso bamboo rhizome bud (Fig. 7). It is of interest that the relevant gene expressions started to drop even at very early stages of underground development, prompting the question how IAA synthesis is regulated and how IAA level is maintained in rhizome buds.

**Fig. 8:**
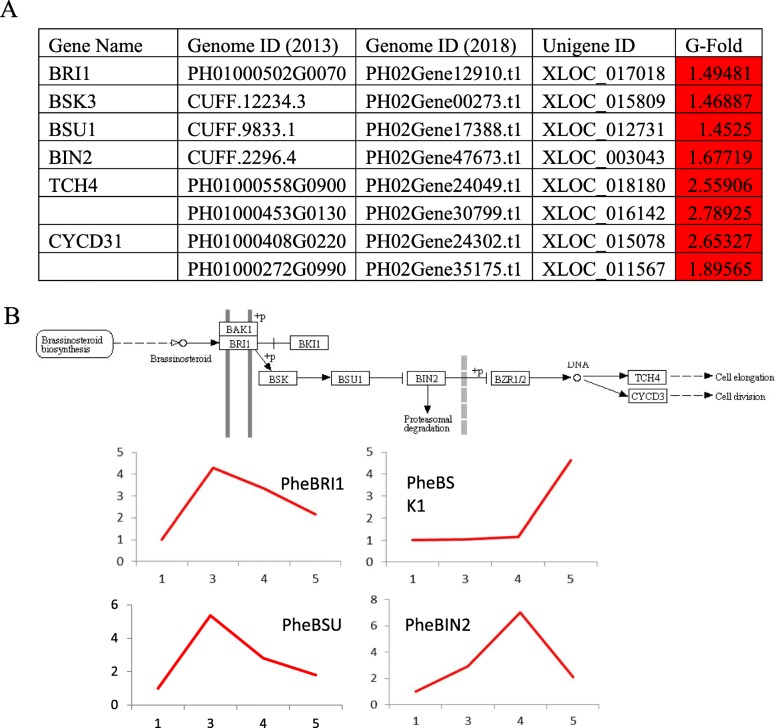
Changes in expression of putative BR signaling pathways genes. **a** Transcriptome analysis of putative genes related to BR synthesis. Red, significantly increased expression at early shoot stage. **b** Relative expression of putative genes in (**a**) at five underground stages of rhizome bud development. Stage 1,3,4 and 5 are germination, development stage 1 and 3, and early shoot stage, respectively. Top, BR signaling pathway derived from Kyoto Encyclopedia of Genes and Genomes Database. The expressions were measured by qPCR and results were normalized based on the germination stage (stage 1) data

BRs are a group of steroidal phytohormones that promote cell growth and stress responses, participating in all stages of plant development. Both our transcriptome analysis data and qPCR quantification showed that the putative genes involved in BR signaling pathway displayed systematic increase in expression in early shoot stages (Fig. 8). Thus, a strong role of BR in early shoot development is indicated.

**Fig. 9:**
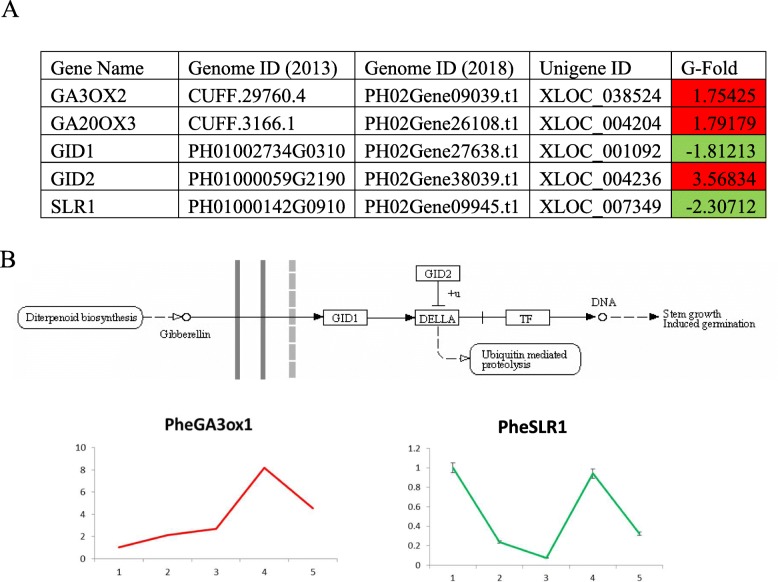
Changes in expression of putative GA signaling pathways genes. **a** Transcriptome analysis of putative genes related to GA signaling. Green, significantly increased expression at germination stage. Red, significantly increased expression at early shoot stage. B Relative expression of putative genes in (**a**) at five underground stages of rhizome bud development. Stage 1 ~ 5 are germination, development stage 1,2 and 3 and early shoot stage, respectively. Top, GA signaling pathway derived from Kyoto Encyclopedia of Genes and Genomes Database. The expressions were measured by qPCR and results were normalized based on the germination stage (stage 1) data

GA stimulates seed germination and meristem development, and in later stages, triggers transition to flowering and fruiting. The transcriptional expression of GA3ox2 and GA20ox3, the key genes responsible for GA synthesis, were greatly elevated at early shoot stage, which is consistent with the trend seen in qPCR results (Fig. 9). The GA signaling activity leads to the decline of DELLA, a protein that inhibits cell growth and promote branching. Plants defective in GA synthesis caused accumulation of DELLA and displayed dwarf and branchy phenotypes. In our study, the DELLA gene expression decreased from germination to early shoot, suggesting an active GA signaling pathway during various underground stages of rhizome bud development (Fig. 9).

### GA stimulates basal internode elongation in Moso bamboo

**Fig. 10:**
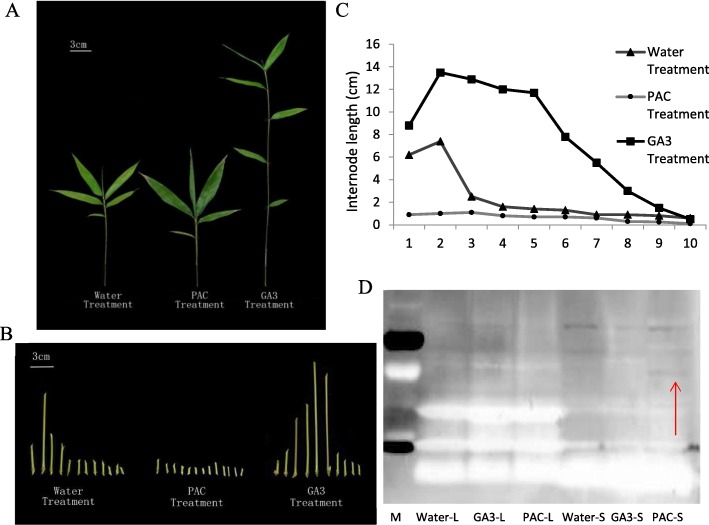
GA stimulate the internode growth in Moso bamboo. **a** Treatment of water (left), PAC (middle) and GA (right) on Moso bamboo seedlings. **b** and **c** Distribution of internode lengths from basal (1) to apical (10) of seedlings. **d** Accumulation of PheDELLA was only detected in stem tissues of PAC treated seedlings. Red arrow, DELLA protein visualized in western blot. M, marker; L, leaves; S, stem. Bars, 3 cm

To confirm our findings from transcriptome analysis, we investigated the effects of GA signaling on fast growth in Moso bamboo. Moso bamboo seedlings were collected from Anji Bamboo Garden and grown in laboratory greenhouse till the establishment of five to seven true leaves before sprayed with GA, the growth inhibitor PAC and water, consecutively for 6 months (Fig. 10a).

The GA treatment significantly boosted the bamboo seedling vertical growth compared to the water treatment control (Fig. 10a, right), consistent with previous study [35]. The PAC control, on the contrary, displayed darker leaves, shorter internode stem length and shorter distance from base to the first true leaf, indicating a slower stem elongation (Fig. 10a, middle). Neither treatment affected bamboo leaf width (LW), leave length (LL) and sheath length (SL) (Fig. S2, A and B).

We further dissected and compared the distribution of internode growth in different treatments (Fig. 10b and c). The water treated wildtype had increased stem length for the first three basal internodes and the stem length sharply decreased as it came closer to the apical side. Treating GA did not disturb the basal stem growth pattern but resulted in longer and more-elongating basal internodes. PAC treatment completely abolished the basal stem growth in all internodes.

DELLA is a critical growth inhibitor during plant development. Same as in other monocot, we only detected one DELLA sequence in *Phyllostachys edulis*, SLR1 (Figure S3). In wildtype *A. thaliana* or DELLA defective mutant *rga*, the DELLA protein level remains undetectable as it is often rapidly degraded. However, an antibody against *A. thaliana* DELLA is able to detect the accumulation of DELLA in *ga3*, a mutant defective in GA synthesis, in Arabidopsis (Fig. S2C). Consistent with our previous findings, the accumulation of DELLA was only observed using the same antibody in PAC treated bamboo stem protein extract where the inhibition of growth was seen (Fig. 10d). Thus, GA and its signaling pathway is actively involved in Moso bamboo fast growth.

## Discussion

To explore the mechanism of *Phyllostachys heterocycla* fast growth from a systematic perspective, we analyzed and compared the transcriptomes of rhizome bud at germination. We identified novel putative genes, alternative splicing and SNPs that would complement current bamboo genomic database. We evaluated the involvement of transcriptional factors, meristem development, carbohydrate metabolism and phytohormones in rapid bamboo rhizome bud development.

### Strong association and high sequence match between transcriptome data and draft genome

Our high throughput sequencing data were mapped to the published bamboo genome with over 93% matching percentage [22]. Such high mapped-reads ratio is probably due to the reason that our sequenced tissues were from the same species, which endowed our reads with strong application to identify new genes and high reliability to examine protein expression level change. However, only less than 40% of the reads were mapped to the annotated regions. The most likely reason was that the tissues we sequenced were different from those used for the annotation of genes previously [22]. As a result, we were able to identify novel putative genes from previous intergenic regions. An improved reference genome assembled in chromosome level was recently released [32]. As the acceleration of sequencing technology and increasing interest on moso bamboo, a more comprehensive understanding of bamboo genome is of inevitable need and will greatly benefit the researchers in the field.

### AS, SNPs and Indels in transcriptome analysis samples

Our AS analysis agreed with previous finding in Arabidopsis that PKinase domain was the most frequently truncated domain family (Class I AS) and WD40 domain were the most likely to be completely inserted/deleted (Class II AS) [47]. The AS class preference of these two domain families could be universal among dicots and monocots and might be derived from their ancestors. Indeed, a recent study showed that AS are more likely to occur in conserved genes. In addition, we found PP2C, DEAD and SNF2_N in Class I and TPR_11 domain, ZF-CCCH domain and IQ domain in Class II were enriched. Splicing variants in SNF2-N and ZF-CCCH family have already been reported in Arabidopsis previously [48, 49]. The high abundance of AS events in moso bamboo rhizome bud speaks volumes of its importance. In this study, we present a preliminary assessment of our RNA-seq data on AS event. How those AS events affect rhizome bud development at various stages is of future interest with the help of a more detailed reference genome [32] and deeper sequencing technology [46].

Natural genomic variations like SNPs and Indels are relatively low in our samples. It is likely due the reason that our samples were collected in the same province of China as the samples used for sequencing the draft genome, which could indicate similar climate, temperature and other environmental factors. We found proteins participating in signal transduction or transcriptional regulation were prone to genomics variations. Those protein families were particularly important for stress response or environmental adaption. Future study is needed to compare the genomic variation in Moso bamboo grown in different regions and their correlation to environmental factors.

### Expression patterns of putative transcription factor, meristem development and carbohydrate metabolism related genes in Moso bamboo rhizome lateral bud

Our transcriptome analysis did not identify all 35 transcription factor families. It is possible that other transcription factor families might specifically express in some other developmental and reproductive stages or be removed from results due to low reliability. The number of TFs highly expressed in germination stage was much more than those in early shoot stage, revealing that the transcription is more activated in germinating rhizome bud. Difference also existed in process functions that enriched in each developmental stage. The ARF, DOF, NAC and WOX family members were enriched in germination stage. These four transcription factor families were actively involved in the plant development. Auxin response factors (ARF) were transcription factors that regulated the expression of auxin response genes [41]. The NAC and WOX transcription factors could participate the regulation of cell division in the early development of primordium [50, 51]. The DOF family participates in the regulation of seed storage protein synthesis in developing endosperm, seed germination, and gibberellin response in post-germinating aleuronic [52]. In this way, the DOF transcription factor might regulate the nutrition which was required by germination rhizome lateral bud to transport from the rhizome and was adjacent over ground culm. On the other hand, the nuclear factor Y subfamily A (NF-YA) heat shock factor (HSF) and MIKC type MADS domain family were only enriched in early shoot stage. Both NF-YA and HSF proteins are important transcriptional factors responsive to abiotic stress, which can be crucial to bamboo shoot dormancy or environmental adaption. The MIKC type MADS domain proteins are essential for plant flowering in floral quartet model. The enrichment of gene expression in this category during early shoot stage may indicate they have addition functions during bamboo growth.

Other putative transcription factors identified in our results such as bHLH protein were enriched in both two developmental stages. The bHLH protein were important regulatory components in transcriptional networks, controlling a diversity of processes from cell proliferation to cell lineage establishment, which was closely related the rhizome lateral bud underground development [53]. Together, our analysis of transcription factors in underground development stage suggested a pool of putative regulatory elements for future functional analysis.

The development of apical and auxiliary meristem in rhizome buds is determinant to bamboo shoot above ground growth. The auxiliary meristem differentiates into sheath, leaves and branches. Our data showed that the auxiliary meristem development related genes were differentially expressed between germination and early shoot stage. We speculated that the auxiliary meristem is not established until later underground bud development due to apical dominance. The apical meristem development may be consistent throughout the rhizome bud underground stages as we did not observe significant changes in expression for genes regulating apical meristem developments.

Carbohydrate metabolism is the main pathway for rhizome bud growth as photosynthesis is not available during underground development. Interestingly, a systematic decline in carbohydrate metabolism activities was observed at early shoot stages. It is possible that more energy and nutrients are needed during germination stages.

### Hormone synthesis and signal transduction regulation in Moso bamboo rhizome lateral bud

In our study, we looked at the dynamics of three hormone signaling pathways in rhizome lateral bud stage and early shoot stage.

IAA synthesis and signaling pathway components were highly conserved across species [24]. We found that the IAA synthesis pathway was highly active at germination stages, coinciding with its important roles in meristem development. Previous research found that the IAA/ABA ratio in praecox rhizome lateral bud regulated the rhizome bud germination [54]. It will be interesting to investigate the activity of abscisic acid pathways during germination stages. Although IAA synthesis were decreased at early shoot stage in our result. It is possible that the polar auxin transport contribution to the IAA levels in early shoot stages beside de novo synthesis. Our analysis did not identify significant changes in auxin signaling pathway. It is likely IAA signaling is consistently active throughout the rhizome bud underground development.

Brassinosteroids (BRs) are steroidal hormones that the regulate plant growth through promoting root cell division and elongation. Consistent with a recent detailed analysis in BR related gene in bamboo [23], the expression of almost all genes in BR signaling pathway were significantly increased in early shoot stage in our data, indicating a potential effect of BRs during rhizome bud development. It is also possible that this elevation is related to the roles of BRs in vascular tissue growth and environmental adaption [55]. Future studies are needed to dissect the effect of BRs during later developmental stages.

The detection of hormones in rhizome lateral bud revealed that GA concentration was high in *Phyllostachys propinqua* shoot [56]. In our results, we found that the GA signaling is increasingly active at later stages of rhizome bud development. PheDELLA (SLR1), whose orthologous in Arabidopsis and Oryza negatively regulate GA signaling pathway, was down-regulated notably in early shoot stage. Consistent with a recent report, GA-treated bamboo seedlings had stimulated internode elongation without disturbing the growth patterns [35]. This pattern could be maintained by meristem development regulation or other presence of other hormones, which requires further research.

DELLA is a critical growth inhibitor during plant development and the GA signaling pathway involves the degradation of DELLA. We observed the accumulation of SLR1 in PAC treated bamboo seedling. Nonspecific interactions were seen on Western blotting can be explained by the use of Arabidopsis specific antibody against *Phyllostachys edulis*. When more tools for Moso bamboo research becomes available, we will be able to dissect the effect of GA on bamboo fast growth better.

## Conclusion

In this study, we utilized high throughput RNA sequencing to analyze the transcriptomes from *Phyllostachys edulis* rhizome lateral buds at two developmental stages. We compared our data to previous genome projects and discovered more than 20,000 novel genes We also identified new AS events, SNPs and Indels, which can serve as markers for genetic studies and hints for gene evolutions. During the course of this study, an updated reference genome for *Phyllostachys edulis* with improved precision and contiguity was published [32], which will greatly benefit the moso bamboo research community. It will be of interest to analyze our RNA-seq results basing on the new reference genome in the future study, especially how AS events and SNPs are differentiated during various stages of lateral rhizome bud development. Analysis on putative genes that differentially expressed between the two developmental stages of rhizome lateral buds shed lights on the roles of transcription factors, carbohydrate metabolisms and phytohormones in Moso bamboo shoot development, as well as a rich list of candidate genes for further functional study. We verified the role of GA in stimulating Moso bamboo internode elongation. Together, our study presented a significant addition to the current genomics resources and valuable source for future studies on bamboo fast growth.

## Methods

### Collection of Moso bamboo rhizome lateral buds

All *Phyllostachys edulies* rhizome lateral buds were collected by Yuting Shou in Anji Bamboo Exposition Garden (119°14′-- 119°53′ E, 30°23′-- 30°53′ N), Zhejiang Province, China during the growing season (Feb. 20th 2010). The sample collection complied with local regulations and did not require specific permission. For high throughput sequencing, the samples for germination stage (MSAJ-01) and early shoot stage (MSAJ-05) are collected underground according to bud length (Fig. 1). For quantitative PCR (Fig. 8), the samples were collected for all underground developmental stages (MSAJ-01 germination; MSAJ-02 development stage 1;MSAJ-03 development stage 2; MSAJ-04 development stage 3; MSAJ-05 early shoot stage). For each developmental stage, three samples from different rhizomes were collected. Fresh tissue from rhizome lateral buds were collected without sheath hair and immediately frozen in liquid nitrogen to avoid RNA degradation. All voucher samples were stored in the laboratory of Dr. Yulong Ding at Nanjing Forestry University in China.

### Construction of cDNA libraries from Moso bamboo lateral buds

Total RNA were extracted from pooled lateral bud samples using the EASYSPIN RNA extraction kit (Yuanpinghao Biotech Co.,Ltd) and treated with RNase-free DNase I for 30 min at 37 °C to remove residual DNA. RNA electrophoresis was run for an initial assessment of sample quality and quantity.

The mRNA was then purified from the total RNA samples and was used to synthesize cDNA. After end repair, adding dA and adapters, the cDNA products were further amplified by PCR using adapter primer sequences. The cDNA libraries were then quantified and sent to sequencing using Illumina Genome Analyzer II.

### Processing RNA-seq data

The distance between the terminals of two paired-end reads is designed around 150 bp, which is shorter than the sum of the lengths of two reads. As a result, a pre-treatment for these reads should be taken before mapping them to the published first draft of bamboo genome [22]. Each pair of reads were firstly merged together into a single read if they shared a sequence with a length longer than 30 bp and an identity higher than 0.9. Each not-merged read was pruned to preserve the high-quality 50 bp part from its terminal.

In order to map the reads, tophat-v2.0.8b was applied with the parameter -g 1 to ensure a unique map result for every read. Cufflinks-v2.1.1 was next used to offer a hybrid annotation to unigenes based on the information from both the first version of bamboo genome annotation [22] and our own mapping results. For Figs. 5, 6, 7, 8 and 9, gene ID from newly published reference genome were obtained [32].

### Discovery of novel transcripts

We firstly picked up genes with all their exons not anchored in the previously annotated genetic regions (class code = ‘u’ in GTF-file produced by Cufflinks). To avoid background noise of sequencing, an expression cutoff should be decided to repel genes with lower expressions. We depicted the distribution of expression of both genetic regions and intergenetic regions in germination stage sample and shoot stage sample (Fig. 2c). The border area between genetic regions and intergentic regions (shadow area in Fig. 2c) was between − 0.2 ~ 0.2 (log10 RPKM). The values − 0.2, 0 and 0.2 were tested and only several ones among thousands of genes were affected. Hence, we set the cutoff as RPKM 1.0.

Trinity-v2013-8-14 was then used to predict ORFs from each transcript of these genes (two or more transcripts may from one gene) and thus produced a list of protein sequences, which were used to blast against annotated *Oryza sativa* proteomics with an e-value cutoff of 1e-5. The transcripts failed to be predicted ORFs were used to blast against Rfam to see whether there exist some ncRNA among these genes. The novel genes with multiple exons were used to make a functional-enrichment analysis.

### Identification of AS events, SNPs and Indels

Astalavista-3.2 was applied to judge alternative splicing events (AS-events) based on the GTF-file produced by Cufflinks. The numbers of different types of AS-events such as intron retention (IR), exon skipping (ES) were counted by AS-code. To learn the impact on protein domain morphogenesis by alternative splicing, we used PfamScan to identify the domain morphogenesis of each protein predicted by Trinity-v2013-8-14 from each isoform.

SNPs between reads from our transcript samples and the genome sequences these reads mapped onto were calculated by inGAP-3-0-1. Only contigs with more than 200 mapped reads were chosen. Those indels with a sequence length shorter than 10 bp were adopted. A functional enrichment analysis was done for high SNPs genes.

### Transcriptome analysis

We implemented Gfold-v1.0.8 to find out differential expression genes in comparing our two Moso bamboo transcript samples. Download the raw data from the Plant-TFDB [57] and KEGG [58] database. The genes with absolute value larger than 1.0 were considered as differential expression genes. Both the lateral bud-up-regulated genes and the shoot-up-regulated genes were respectively used to make a functional enrichment analysis.

### Functional annotation and enrichment analysis

We first blasted all the proteins predicted by trinity from the whole transcripts to the proteins of *Oryza sativa* downloaded from RAPDB with an e-value cutoff of 1e-5. With the RAPDB version of *Oryza sativa* mapping list downloaded from Mapman as functional annotation system, we then pick up the target protein with the lowest e-value among every protein from every isoform of one gene and used the Mapman’s annotation of the target protein to predict the functional category of this gene.

To make functional enrichment, both genes from control set and background set were counted in each category and the genes assigned in one category were also counted in its parent category, due to the hierarchical structure of Mapman’s annotation. Then of each rank, a fisher exact test was taken on the categories. On the functional enrichment analysis of differential expressed genes and differential expressed isoforms, all the genes found by Cufflinks were taken as the background gene set, whereas on the analysis of SNPs, only the annotated genes in the published bamboo genome were used.

### Hormone related gene expression by quantitative PCR

Total RNA extracted from each underground stage sample were reverse transcribed to cDNA. Depending on sample availability, selected putative genes expressions were quantified in at least four underground stages using gene specific primers in qPCR (Table S1). Moso bamboo actin was used as internal reference. The PCR program included: 94 °C for 5 min, 35 cycles of 94 °C for 30 s, 54 °C for 30 s and 72 °C for 1 min, and 72 °C for 10 min.

### Bamboo seedling growth test

Before growth test, Moso bamboo seedlings were grown in pots in greenhouse conditions until 5 ~ 7 true leaves were established. The seedlings were then separated into three groups and sprayed with water, GA and PAC for about 180 days, respectively. After treatment, the growth of seedlings in each group were recorded and compared.

### Western blotting to detect DELLA (SLR1) protein

0.1 g of each sample from the growth test above were collected in liquid nitrogen and grinded before adding 300 μl protein extraction buffer (50 mM Tris-HCl pH 8.0, 150 mM NaCl, 1% Triton-X 100 and 100 μg/ml PMSF). The samples were mixed on ice for 30 min and then boiled at 100 °C for 10 min. The crude extract was then centrifuged at 12000 rpm for 30 min. 10 μl of the supernatant were mixed with standard SDS loading buffer and loaded onto an SDS-PAGE gel. After electrophoresis, the samples were transferred to PVDF membrane and sequentially blotted with anti-DELLA antibody (1:3750) (ref) and secondary antibody (1:5000) in 5% NFDM-TBST solution. The results of western blotting were revealed by chemifluorescence.

## Supplementary information

**Additional file 1: Fig. S1** Differential gene expression in two underground development stages of moso bamboo. **Fig. S2** Effects of GA treatment on bamboo seedlings. (A) Illustration of leave and sheath of seedling under water (left), PAC (middle) and GA (right) treatment. Bar, 3 cm. (B) Comparisons of leave length (LL), leave width (LW), sheath length (SL) and internode length (IL) during growth test. (C) AtDELLA detection in Arabidopsis strains through western blotting. Col, wildtype; *ga3*, GA synthesis deficient mutant; *rga*, DELLA mutant. Red arrow, protein visualized in western blot. **Fig. S3** ClustalW alignment of SLR1 sequences in RNA-seq, Peng et al. [22], Zhao et al. [32] and *Oryza Savita.* The consensus region was noted by *.

**Additional file 2: Table S1.** Primer used in quantitative PCR.

## Data Availability

The RNA-seq data (GSE142030) for the current study are available on GEO.

## Abbreviations

AS: Alternative splicing
BR: Brassinolides
GA: Gibberellic acids
SNP: Single nucleotide polymorphism
MEG: Multiple exon genes
SEG: Single exon genes
IAA: Indole-3-acettic acid
TCA: Tri-citric acid
PPP: Pentose phosphate pathway
RPKM: Reads per kilobase million

## References

[1] Beveridge CA, Mathesius U, Rose RJ, Gresshoff PM (2007). Common regulatory themes in meristem development and whole-plant homeostasis. Curr Opin Plant Biol.

[2] Fu J (2001). Chinese moso bamboo: its importance. Bamboo..

[3] Erfa ZYHWQ, Liguang C. Hormone content and distribution in *Phyllostachys heterocycla cv. pubescens* during period of shoot emergence. Sci Silvae Sin. 1998;S1..

[4] He X-Q, Suzuki K, Kitamura S, Lin J-X, Cui K-M, Itoh T (2002). Toward understanding the different function of two types of parenchyma cells in bamboo culms. Plant Cell Physiol.

[5] Li X, Shupe T, Peter G, Hse C, Eberhardt T (2007). Chemical changes with maturation of the bamboo species *Phyllostachys pubescens*. J Trop For Sci.

[6] Yu Y, Tian G, Wang H, Fei B, Wang G (2011). Mechanical characterization of single bamboo fibers with nanoindentation and microtensile technique. Holzforschung..

[7] Lee C, Chin T (1960). Comparative anatomical studies of some Chinese bamboos. Acta Bot Sin.

[8] Wang Y, Li J (2008). Molecular basis of plant architecture. Annu Rev Plant Biol.

[9] Magel E, Kruse S, Lütje G, Liese W (2005). Soluble carbohydrates and acid invertases involved in the rapid growth of developing culms in *Sasa palmata* (bean) Camus. Bamboo Sci Cult.

[10] Calvo S, Lovison G, Pirrotta M, Di Maida G, Tomasello A, Sciandra M (2006). Modelling the relationship between sexual reproduction and rhizome growth in *Posidonia oceanica* (L.) Delile. Mar Ecol.

[11] Bateman RM, Crane PR, DiMichele WA, Kenrick PR, Rowe NP, Speck T (1998). Early evolution of land plants: phylogeny, physiology, and ecology of the primary terrestrial radiation. Annu Rev Ecol Evol Syst.

[12] McIntyre GI (1964). Influence of nitrogen nutrition on bud and rhizome development in *Agropyron repens* L. Beauv. Nature.

[13] McIntyre GI (1976). Apical dominance in the rhizome of *Agropyron repens*: the influence of water stress on bud activity. Can J Bot.

[14] McIntyre GI (1965). Some effects of the nitrogen supply on the growth and development of *acropyron repens* l. beauv.*. Weed Res.

[15] McIntyre GI (1987). Studies on the growth and development of *Agropyron repens*: interacting effects of humidity, calcium, and nitrogen on growth of the rhizome apex and lateral buds. Can J Bot.

[16] Leakey R, Chancellor R, Vince-Prue D (1978). Regeneration from rhizome fragments of *Agropyron repens* (L.) Beauv. IV. Effects of light on bud dormancy and development of dominance amongst shoots on multi-node fragments. Ann Bot.

[17] Wang K, Peng H, Lin E, Jin Q, Hua X, Yao S (2010). Identification of genes related to the development of bamboo rhizome bud. J Exp Bot.

[18] Liu HL, Wu M, Li F, Gao YM, Chen F, Xiang Y (2018). TCP transcription factors in moso bamboo (*Phyllostachys edulis*): genome-wide identification and expression analysis. Front Plant Sci.

[19] Pan Feng, Wu Min, Hu Wenfang, Liu Rui, Yan Hanwei, Xiang Yan (2019). Genome-Wide Identification and Expression Analyses of the bZIP Transcription Factor Genes in moso bamboo (Phyllostachys edulis). International Journal of Molecular Sciences.

[20] Shi Y, Liu H, Gao Y, Wang Y, Wu M, Xiang Y (2019). Genome-wide identification of growth-regulating factors in moso bamboo (*Phyllostachys edulis*): in silico and experimental analyses. Peer J.

[21] Hu F, Wang D, Zhao X, Zhang T, Sun H, Zhu L (2011). Identification of rhizome-specific genes by genome-wide differential expression analysis in *Oryza longistaminata*. BMC Plant Biol.

[22] Peng Z, Lu Y, Li L, Zhao Q, Feng Q, Gao Z (2013). The draft genome of the fast-growing non-timber forest species moso bamboo (*Phyllostachys heterocycla*). Nat Genet.

[23] Wang S, Sun H, Xu X, Yang K, Zhao H, Li Y (2019). Genome-wide identification and expression analysis of brassinosteroid action-related genes during the shoot growth of moso bamboo. Mol Biol Rep.

[24] Wang W, Gu L, Ye S, Zhang H, Cai C, Xiang M (2017). Genome-wide analysis and transcriptomic profiling of the auxin biosynthesis, transport and signaling family genes in moso bamboo (*Phyllostachys heterocycla*). BMC Genomics.

[25] Ye J, Zhang Y, Fu Y, Zhou M, Tang D (2019). Genome-wide identification and expression analysis of gibberellin biosynthesis, metabolism and signaling family genes in *Phyllostachys edulis*. Chin J Biotechnol.

[26] He CY, Cui K, Zhang JG, Duan AG, Zeng YF (2013). Next-generation sequencing-based mRNA and microRNA expression profiling analysis revealed pathways involved in the rapid growth of developing culms in moso bamboo. BMC Plant Biol.

[27] Light S, Elofsson A (2013). The impact of splicing on protein domain architecture. Curr Opin Struct Biol.

[28] Kriventseva EV, Koch I, Apweiler R, Vingron M, Bork P, Gelfand MS (2003). Increase of functional diversity by alternative splicing. Trends Genet.

[29] Keren H, Lev-Maor G, Ast G (2010). Alternative splicing and evolution: diversification, exon definition and function. Nat Rev Genet.

[30] Finet C, Berne-Dedieu A, Scutt CP, Marlétaz F (2013). Evolution of the ARF gene family in land plants: old domains, new tricks. Mol Biol Evol.

[31] Chung HS, Howe GA (2009). A critical role for the TIFY motif in repression of jasmonate signaling by a stabilized splice variant of the JASMONATE ZIM-domain protein JAZ10 in *Arabidopsis*. Plant Cell.

[32] Zhao H, Gao Z, Wang L, Wang J, Wang S, Fei B (2018). Chromosome-level reference genome and alternative splicing atlas of moso bamboo (*Phyllostachys edulis*). Gigascience.

[33] Li L, Shi Q, Hou D, Cheng Z, Li J, Ma Y, Li X, Mu S, Gao J (2018). Transcriptome analysis of alternative splicing in different moso bamboo tissues. Acta Physiol Plant.

[34] Li L, Hu T, Li X, Mu S, Cheng Z, Ge W (2016). Genome-wide analysis of shoot growth-associated alternative splicing in moso bamboo. Mol Gen Genomics.

[35] Zhang H, Wang H, Zhu Q, Gao Y, Zhao L, Wang Y (2018). Transcriptome characterization of moso bamboo (*Phyllostachys edulis*) seedlings in response to exogenous gibberellin applications. BMC Plant Biol.

[36] Wang XQ, Zhao L, Eaton DA, Li DZ, Guo ZH (2013). Identification of SNP markers for inferring phylogeny in temperate bamboos (*Poaceae: Bambusoideae*) using RAD sequencing. Mol Ecol Resour.

[37] Hu C, Jin A, Zhang Z (1995). Change of Endohormone in mixed bud on lei bamboo rhizome during differentiation. J Zhejiang For College.

[38] Zhang Z, Hu C, Jin A (1995). Observation of the morphology and the structure of *Phyllostachys praecox* rhizome lateral bud developing into shoot. J Bamboo Res.

[39] Roberts A, Pimentel H, Trapnell C, Pachter L (2011). Identification of novel transcripts in annotated genomes using RNA-Seq. Bioinformatics..

[40] Grabherr MG, Haas BJ, Yassour M, Levin JZ, Thompson DA, Amit I (2011). Full-length transcriptome assembly from RNA-Seq data without a reference genome. Nat Biotechnol.

[41] Guilfoyle TJ, Hagen G (2007). Auxin response factors. Curr Opin Plant Biol.

[42] Burge SW, Daub J, Eberhardt R, Tate J, Barquist L, Nawrocki EP (2013). Rfam 11.0: 10 years of RNA families. Nucleic Acids Res.

[43] Yan H, Dai X, Feng K, Ma Q, Yin T (2016). IGDD: a database of intronless genes in dicots. BMC Bioinformatics.

[44] Yan H, Zhang W, Lin Y, Dong Q, Peng X, Jiang H (2014). Different evolutionary patterns among intronless genes in maize genome. Biochem Biophys Res Commun.

[45] Foissac S, Sammeth M (2007). ASTALAVISTA: dynamic and flexible analysis of alternative splicing events in custom gene datasets. Nucleic Acids Res.

[46] Wang T, Wang H, Cai D, Gao Y, Zhang H, Wang Y (2017). Comprehensive profiling of rhizome-associated alternative splicing and alternative polyadenylation in moso bamboo (*Phyllostachys edulis*). Plant J.

[47] Wang H, You C, Chang F, Wang Y, Wang L, Qi J (2014). Alternative splicing during *Arabidopsis* flower development results in constitutive and stage-regulated isoforms. Front Genet.

[48] Addepalli B, Hunt AG (2008). Ribonuclease activity is a common property of *Arabidopsis* CCCH-containing zinc-finger proteins. FEBS Lett.

[49] Archacki R, Sarnowski TJ, Halibart-Puzio J, Brzeska K, Buszewicz D, Prymakowska-Bosak M (2009). Genetic analysis of functional redundancy of BRM ATPase and ATSWI3C subunits of *Arabidopsis* SWI/SNF chromatin remodelling complexes. Planta..

[50] Fang Y, You J, Xie K, Xie W, Xiong L (2008). Systematic sequence analysis and identification of tissue-specific or stress-responsive genes of NAC transcription factor family in rice. Mol Genet Genom Med.

[51] van der Graaff E, Laux T, Rensing SA (2009). The WUS homeobox-containing (WOX) protein family. Genome Biol.

[52] Lijavetzky D, Carbonero P, Vicente-Carbajosa J (2003). Genome-wide comparative phylogenetic analysis of the rice and *Arabidopsis* Dof gene families. BMC Evol Biol.

[53] Toledo-Ortiz G, Huq E, Quail PH (2003). The *Arabidopsis* basic/helix-loop-helix transcription factor family. Plant Cell.

[54] Huang J, Zhang B, Liu L, Qiu L (2001). Dynamic changes of endophytohormones in rhizomal buds of *Phyllostachys praecox*. Sci Silvae Sin.

[55] Ye H, Li L, Yin Y (2011). Recent advances in the regulation of brassinosteroid signaling and biosynthesis pathways. J Integr Plant Biol.

[56] Zhihong SXCXX, Zhihua WXSZP (1996). Study on early shooting, high-yield and keeping mother bamboo of *Phyllostachys propinqua*. J Zhejiang For Sci Technol.

[57] Jin J, Zhang H, Kong L, Gao G, Luo J (2014). PlantTFDB 3.0: a portal for the functional and evolutionary study of plant transcription factors. Nucleic Acids Res.

[58] Kanehisa M, Goto S (2000). KEGG: Kyoto encyclopedia of genes and genomes. Nucleic Acids Res.

