# Supplementary figures and images for: Transcriptome analysis of lateral buds from Phyllostachys edulis rhizome during germination and early shoot stages
Source: BMC Plant Biol. 2020 May 24;20:229. doi: 10.1186/s12870-020-02439-8 (PMC7245953; doi:10.1186/s12870-020-02439-8)

Figure S1

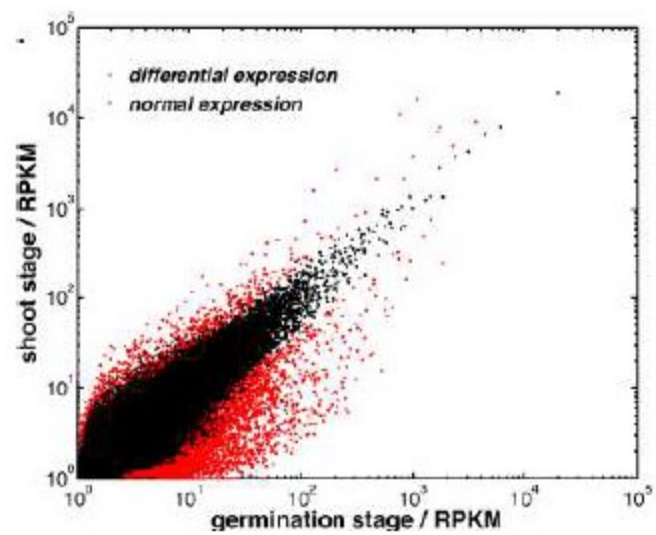

Figure S2

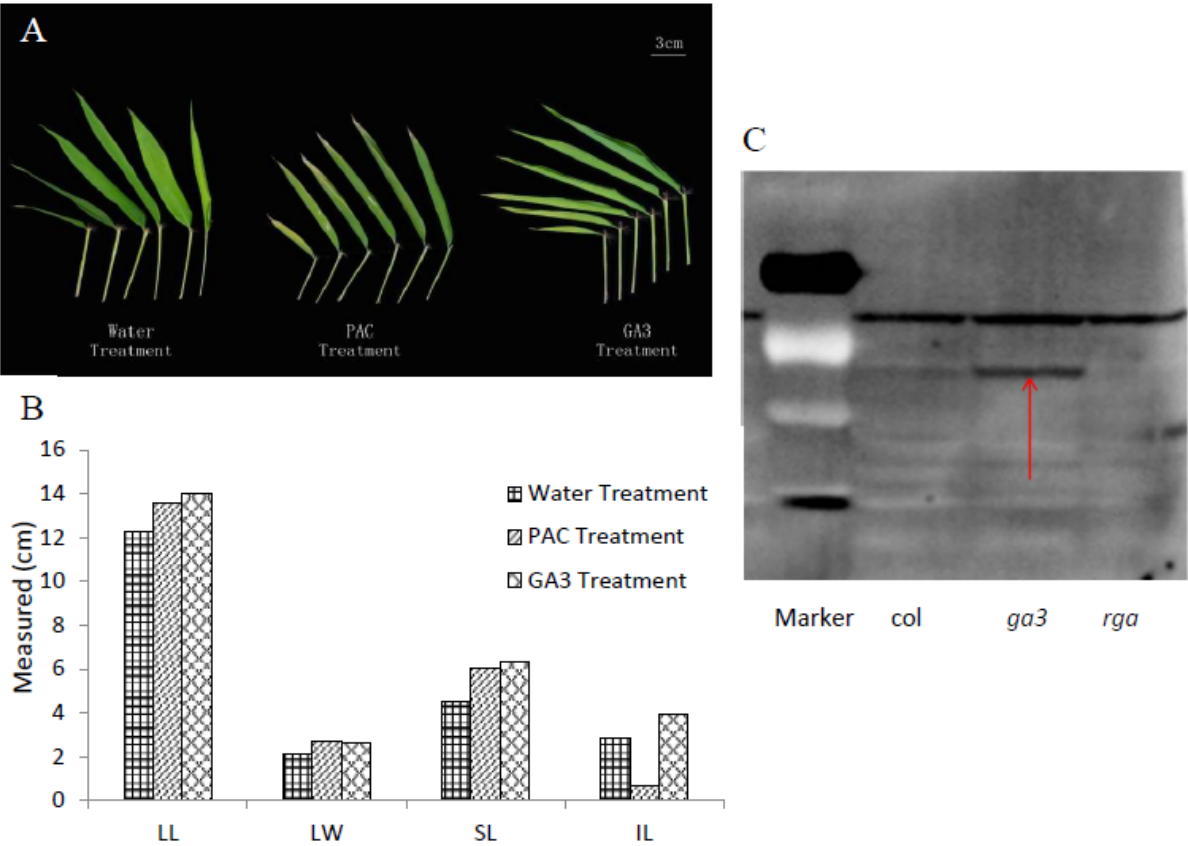

Figure S3

[illegible]

Supplement: Supplementary file 1 — Additional file 1: Fig. S1 Differential gene expression in two underground development stages of moso bamboo. Fig. S2 Effects of GA treatment on bamboo seedlings. (A) Illustration of leave and sheath of seedling under water (left), PAC (middle) and GA (right) treatment. Bar, 3 cm. (B) Comparisons of leave length (LL), leave width (LW), sheath length (SL) and internode length (IL) during growth test. (C) AtDELLA detection in Arabidopsis strains through western blotting. Col, wildtype; ga3, GA synthesis deficient mutant; rga, DELLA mutant. Red arrow, protein visualized in western blot. Fig. S3 ClustalW alignment of SLR1 sequences in RNA-seq, Peng et al. [22], Zhao et al. [32] and Oryza Savita. The consensus region was noted by *. [file 12870_2020_2439_MOESM1_ESM.pdf]
