# Supplementary material for: Transcriptome analysis of lateral buds from Phyllostachys edulis rhizome during germination and early shoot stages
Source: BMC Plant Biol. 2020 May 24;20:229. doi: 10.1186/s12870-020-02439-8 (PMC7245953; doi:10.1186/s12870-020-02439-8)
Supplement: Supplementary file 2 — Additional file 2: Table S1. Primer used in quantitative PCR. [file 12870_2020_2439_MOESM2_ESM.docx]

**Table S1** Primer used in quantitative PCR

| Unigene | Forward primer 5’- 3’ | Reverse primer 5’- 3’ |
| --- | --- | --- |
| Pe-Actin | GGTGTGAGCCATACTGTGCCCAT | TTTCCCGTTCAGCAGAGGTTGTG |
| AMI | TTCTGAGGCTACCTTATGCTGAT | TTTGAGTCCCCATTCTTGTGTC |
| CYP79B2 | TCAAGACTATTGTCAACAACGGG | TCTGAGAGTGAAAAGTGCATCCA |
| CYP79B3 | CTTCCAAAACCTCCCCTACC | CCCACACGTTCACCATGAC |
| TAA1 | GGACGACATCTTCGCCTTC | CCTCTCGCATTTCACCCAC |
| YUCC | GCTTCCCCTTGTGCCTTTC | TCCACTTCCTCCCCCATTC |
| GA3ox1 | ATCACTCGGATACTTCCTCGGCC | CCGTGGAGACCATCTTGAGCG |
| SLR1 | GTGTGATGAGATGGACAATGGA | AAGAGCAGGGAGCAGCATTC |
| BRI1 | GGGACTCTTGTTCTCGCTGTT | TTGCCTCTTCATCCTTCTGCT |
| BSK1 | GTGCGATTAGCTTCAAGGTG | CCATCAGTTCGTAAGAGGGAG |
| BSU1 | CTCGCCATACAGCACTAACAGC | CCAAGGGTGGGAAATCAAAA |
| BIN2 | CATGAACCAAAGGATGCCACT | CTCCAGGTACAGTATGAATGTAAGC |
